# Comparison of autonomic stress reactivity in young healthy versus aging subjects with heart disease

**DOI:** 10.1371/journal.pone.0216278

**Published:** 2019-05-08

**Authors:** Nil Z. Gurel, Andrew M. Carek, Omer T. Inan, Oleksiy Levantsevych, Naser Abdelhadi, Muhammad Hammadah, Wesley T. O’Neal, Heval Kelli, Kobina Wilmot, Laura Ward, Steven Rhodes, Brad D. Pearce, Puja K. Mehta, Michael Kutner, Ernest Garcia, Arshed Quyyumi, Viola Vaccarino, Paolo Raggi, J. Douglas Bremner, Amit J. Shah

**Affiliations:** 1 School of Electrical and Computer Engineering, Georgia Institute of Technology, Atlanta, Georgia, United States of America; 2 Rollins School of Public Health, Emory University, Atlanta, Georgia, United States of America; 3 Department of Medicine, Division of Cardiology, Emory University School of Medicine, Atlanta, Georgia, United States of America; 4 Department of Biostatistics & Bioinformatics, Rollins School of Public Health, Emory University, Atlanta, Georgia, United States of America; 5 Department of Radiology, Emory University School of Medicine, Atlanta, Georgia, United States of America; 6 Mazankowski Alberta Heart Institute, University of Alberta, Edmonton, Canada; 7 Department of Psychiatry, Emory University School of Medicine, Atlanta, Georgia, United States of America; University of Illinois at Urbana-Champaign, UNITED STATES

## Abstract

**Background:**

The autonomic response to acute emotional stress can be highly variable, and pathological responses are associated with increased risk of adverse cardiovascular events. We evaluated the autonomic response to stress reactivity of young healthy subjects and aging subjects with coronary artery disease to understand how the autonomic stress response differs with aging.

**Methods:**

Physiologic reactivity to arithmetic stress in a cohort of 25 young, healthy subjects (< 30 years) and another cohort of 25 older subjects (> 55 years) with CAD was evaluated using electrocardiography, impedance cardiography, and arterial pressure recordings. Stress-related changes in the pre-ejection period (PEP), which measures sympathetic activity, and high frequency heart rate variability (HF HRV), which measures parasympathetic activity, were analyzed as primary outcomes.

**Results:**

Mental stress reduced PEP in both groups (p<0.01), although the decrease was 50% greater in the healthy group. Mean HF HRV decreased significantly in the aging group only (p = 0.01).

**Discussion:**

PEP decreases with stress regardless of health and age status, implying increased sympathetic function. Its decline with stress may be attenuated in CAD. The HF HRV (parasympathetic) stress reactivity is more variable and attenuated in younger individuals; perhaps this is related to a protective parasympathetic reflex.

**Trial registration:**

ClinicalTrials.gov Identifier: NCT02657382.

## Introduction

Cardiovascular and behavioral responses to emotional stress have been associated with increased risk of primary [1–4] and secondary [5–8] coronary artery disease events. Furthermore, acute emotional stress events may trigger myocardial infarction and sudden cardiac death, but the mechanisms are not clear. In some studies, exaggerated mental stress reactivity is associated with increased future risk [9]. Paradoxically, blunted stress reactivity is also associated with increased risk [10]. This underscores the need for more research on the stress response in young, healthy groups and aging groups with CAD.

Prior studies have focused on stress-related changes in heart rate (HR) and blood pressure (BP) in healthy subjects [11–15]. A subset of studies have also evaluated stress-related reactivity of specific cardiac markers, including ejection fraction (EF) and myocardial blood flow (MBF) in patients with CAD [16–21]. The limitation of these metrics is that they do not provide specific autonomic pathways that would yield insight on targeted interventions. Studies which examine the physiologic measures specific to both the sympathetic nervous system (SNS) and parasympathetic nervous system (PNS) changes with mental stress may provide more insight with regards to possible receptor-targeted therapeutics [8, 22].

The high frequency component of heart rate variability (HF HRV), computed based on beat-to-beat interval (R-R interval) variations on the electrocardiogram (ECG), is one of the most commonly studied ways of non-invasively assessing PNS activity [23–25]. During emotional stress, PNS withdrawal often occurs, which is usually accompanied by decreased HF HRV [23]. The pre-ejection period (PEP), measured by the time delay between the onset of electrical depolarization (QRS waveform) and aortic valve opening, is considered as a non-invasive assessment of cardiac SNS activity [26, 27]. Specifically, decreased PEP is a surrogate measure of increased cardiac contractility, which may change acutely due to autonomic influences. In particular, decreased PEP reflects increased cardiac β_1_ receptor stimulation [26].

In this study, we examined two separate psychophysiology experiments with similar protocols to assess the stress reactivity in two different cohorts to evaluate the influence of age and coronary disease on stress reactivity. Aging and coronary disease are considered synonymous as they are strongly correlated: CAD is a manifestation of accelerated aging [28]. We consider aging as a progressive deterioration of physiological function, defined by a spectrum with two extremities: young, healthy subjects on one end, and aging patients with heart disease on another end. As CAD is an accelerator of biological age, aging patients with CAD and young, healthy subjects will represent the extreme ends of the aging spectrum. One cohort consisted of young (<30 years), healthy subjects, and the other cohort consisted of older (>55 years) subjects with CAD. In both cases, we examined stress reactivity to laboratory-induced arithmetic mental stress using SNS (PEP) and PNS (HF HRV) specific metrics. Additional standard metrics such as BP and HR were also assessed. We hypothesized that both PEP and HF HRV would decrease with mental stress in both groups to indicate sympathetic activation and parasympathetic inhibition, and the results would be exaggerated in the aging cohort vs. the healthy cohort as an indication of their worsened health status.

## Methods

### Study groups

#### Young healthy group

The study was performed under a protocol approved by the Georgia Institute of Technology Institutional Review Board (#H13512), at Georgia Institute of Technology, Atlanta, GA, between 01/07/2016 to 06/18/2016. A total of 25 adults (mean ± SD 25.4 ± 4.4 years, 5 females) were recruited through referral and written, informed consent was obtained. The subjects were students or staff without clinically apparent CAD or traditional risk factors for CAD.

Each healthy control subject was asked to relax for 15 minutes in a quiet, temperature-controlled (22°C to 24°C) room, after which the baseline resting signals were obtained. Mental stress was induced by a one-minute mental arithmetic test with serial addition paradigm, by the research staff. Arithmetic stressor was chosen as it requires active coping, and known to induce cardiovascular changes the most, compared to other types of mental stressors [29, 30]. Negative feedback was provided for incorrect answers and delayed response times, as is standard protocol for mental stress testing [19, 31].

Physiologic monitoring was conducted with 3-lead ECG, impedance cardiogram (ICG) and continuous BP during rest, stress, and recovery. ECG and ICG signals were collected using BN-EL50 and BN-NICO Amplifiers, Biopac Systems (Goleta, CA). BP was collected using Finapres (Enchede, Netherlands). All signals from the group of healthy subjects were collected at 2kHz sampling rate.

#### Aging group with CAD

**Fig 1:**
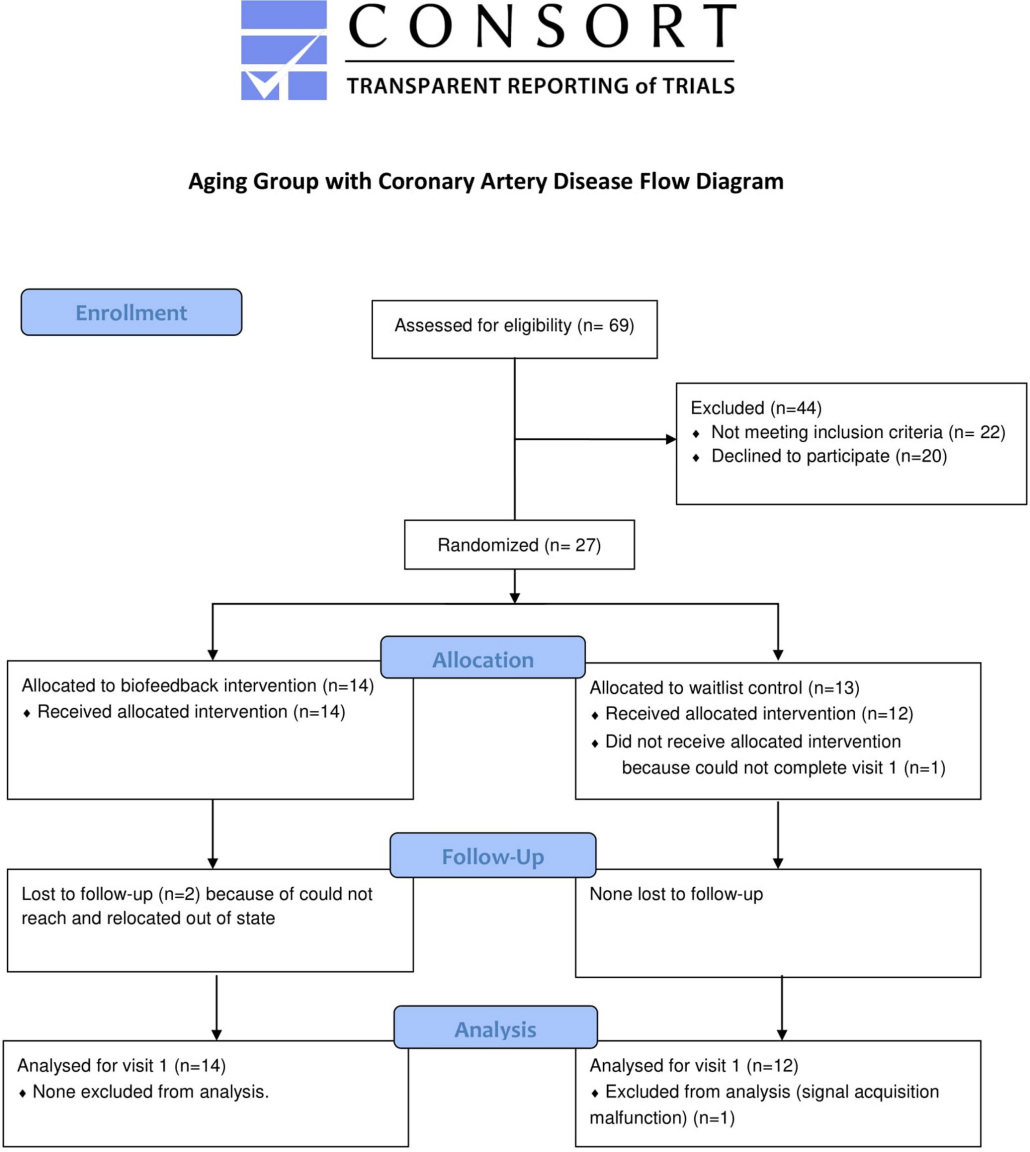
CONSORT diagram for aging group with CAD.

A similar protocol to the healthy subjects was performed in a separate study, “Biofeedback in Mental Stress Ischemia” (ClinicalTrials.gov #NCT02657382), at Emory University School of Medicine, Atlanta, GA, between 03/30/2016 to 03/29/2017. A total of 25 adults (mean ± SD 64.8±5.9 years, 8 females) were recruited from the “Mental Stress Ischemia Mechanisms and Prognosis” cohort for a randomized study evaluating biofeedback vs. waitlist control, and written, informed consent was obtained [32]. Details of the inclusion/exclusion criteria can be found elsewhere [32], but briefly, subjects with any history of CAD based on abnormal angiogram (>20% stenosis), nuclear stress test, or myocardial infarction were enrolled. Fig 1 shows the CONSORT diagram of this group.

Comorbidities, medical history and medication usage of this cohort were listed in Table 1. Subjects who use beta-blockers were asked to hold usage in the morning before the procedure. As part of their evaluation in the Biofeedback study, mental stress reactivity was assessed during three separate visits, including pre- intervention (vs. waitlist), post-intervention (vs. waitlist) at 8 weeks, and after waitlist intervention period (16 weeks), in which the wait-listed subjects received the intervention. Only the first per subject visit was evaluated for this analysis to allow for independent sampling.

**Table 1:** Aging cohort medical history.

Comorbidities	%	History	%	Medication Usage	%
**Abnormal Angiogram**	60%	Hypertension	68%	Beta-blockers	68%
**PTCA**	72%	Diabetes	40%	Antidepressants	20%
**CABG**	28%	Dyslipidemia	80%	ACE inhibitors	40%
**Myocardial Infarction**	36%	Smoking	48%		

Values show the percentages of the population (n = 25). Subjects were asked to hold beta-blockers during the study. Abbreviations: CABG, coronary artery bypass grift; PTCA, percutaneous transluminal coronary angioplasty; ACE: Angiotensin converting enzyme.

Similar to the healthy group study, each aging group subject was asked to relax in a temperature-controlled room. A three-minute math serial subtraction paradigm was conducted with negative feedback, by the research staff. ECG and ICG were collected using the VU AMS Ambulatory Monitoring System (Amsterdam, Netherlands), at a sampling rate of 1 kHz. Non-continuous systolic/diastolic BP values were collected using an automatic Omron blood pressure monitor in every 0, 5, 25 and 30 minutes of rest and each one minute of stress.

For this group of subjects, the relationship of CAD severity and stress reactivity was also quantified using Gensini scores on their last angiogram. The Gensini scores quantify CAD burden from the extent and severity of the coronary artery involvement [33, 34]. The Gensini scores were computed by assigning a severity score to each coronary stenosis according to the degree of luminal narrowing and its importance based on location. Linear models estimated the change in physiological measures with stress that corresponded to the Gensini scores for each respective subject.

### Signal processing and feature extraction

**Fig 2:**
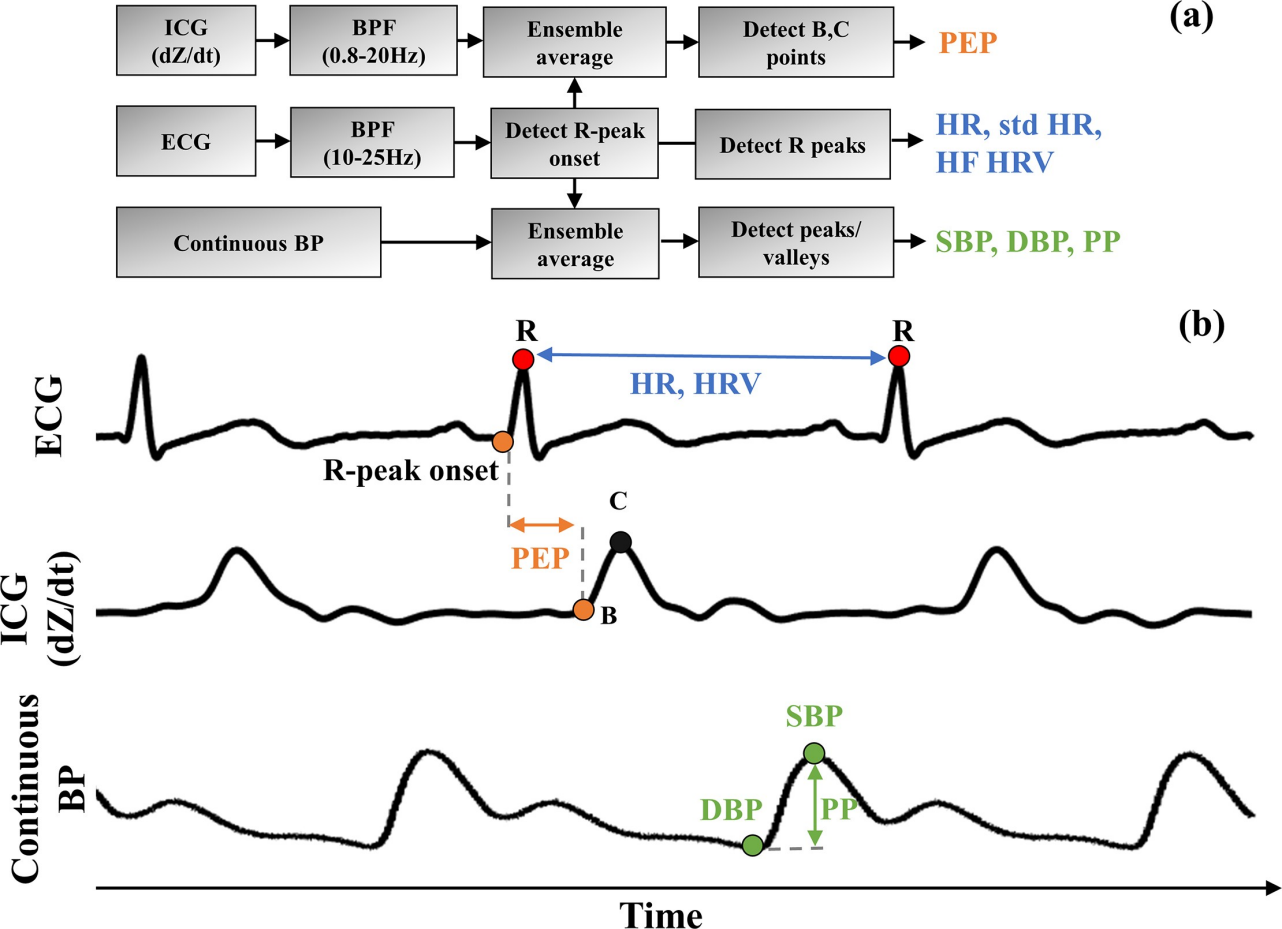
**a) Signal processing steps.** ICG and Continuous BP signals were segmented using the R-peak onset of ECG signal. Signal processing algorithms described in text were used for feature extraction for each interval (rest and stress). ICG: Impedance cardiography, ECG: Electrocardiography, Continuous BP: Continuous blood pressure, BPF: Bandpass filter, PEP: Pre-ejection period, HR: Heart rate, std HR: Standard deviation of HR, HF HRV: High-frequency heart rate variability, SBP: Systolic Blood Pressure, DBP: Diastolic Blood Pressure, PP: Pulse pressure. **b) Waveforms showing extracted features.** HR was calculated using R-R intervals of ECG. PEP was calculated as the time interval between the R-onset of ECG and ICG B-point. SBP and DBP were calculated by detecting the global maximum and minimum of continuous BP beats, respectively.

Fig 2A summarizes the signal processing steps and Fig 2B shows the extracted physiological markers. All signals were processed using MATLAB 2016a (Natick, MA). The ECG was bandpass filtered (10–25 Hz, linear, finite impulse response (FIR)) to exaggerate QRS complex for feature extraction and noise reduction. Similarly, the ICG (dZ/dt) was bandpass filtered (0.8-20Hz, linear, FIR) for noise reduction. Since all subjects had normal sinus rhythm, ECG R-peaks were detected with thresholding to calculate HR and HRV, and edited, if needed, for erroneous peak detection due to noise. For HRV, the non-constant R-R intervals were interpolated using piecewise cubic hermite interpolating polynomial to obtain constant intervals between R-peaks, to be able to compute power spectral density (PSD). The area under the PSD curve from 0.15 Hz-0.4 Hz was extracted as HF HRV [23]. The standard deviation of HR (std HR) was also calculated as a secondary measure.

PEP measurement started with the detection of the onset of myocardial depolarization. This onset was used as a reference for the beginning of each myocardial heartbeat cycle. The filtered ECG signal exaggerated the onset of R-peak and S-peaks, such that the QRS complex was distinguishable with two negative peaks that correspond to R-peak onset and S-peak, respectively. To detect the onset, first the filtered ECG signal was inverted, and both R-peak onset and S-peak were found by thresholding (two peaks per beat). Then, the odd-numbered peaks were selected, which correspond to the onset. Manual review of peak detection was also performed to exclude areas with artifact. The R-peak onsets were used to segment all the signals into individual beats.

To reduce the effects of motion artifacts on the individual, segmented ICG beats, exponential moving averaging of successive beats was implemented [35]. Since the underlying signal properties are changing with time, exponentially decreasing weighting gives more emphasis to the more recent beats, while still providing noise reduction based on the averaging. We determined that a 10-beat time constant for ICG for exponential weighting was long enough to reduce motion artifacts and short enough such that the changes in signal properties over time associated with the mental stress were preserved [36].

PEP was calculated as the difference between the onset of myocardial depolarization and the opening of aortic valve. The B-point on the dZ/dt waveform corresponds to the opening of aortic valve, however, its mathematical detection is not well-defined in the literature. Although it is a physiological event occurring in every single cardiac cycle, the notch or inflection point defining the B-point is not always apparent [37]. Several mathematical algorithms have been developed for the automatic estimation of B-point [26, 37, 38], however there are discrepancies between methods in different experimental conditions [39]. Recently, three popular B-point detection algorithms have been compared, and the algorithm based on the third derivative of ICG was validated to perform significantly better [40]. Therefore, in this work, the third-derivative approach was used to detect B-point. Specifically, the global maxima (dZ/dt_max_) was first calculated (C-point), which represents maximum left ventricular flow [26]. Then, the maximum of the third derivative (dZ^3^/dt^3^) of the ICG signal before the C-point was calculated as the B-point. Therefore, PEP was calculated as the difference between the ECG myocardial depolarization onset and ICG B-point.

Continuous BP signal was not smoothed further, since BP device applies post-processing. Continuous BP physiological markers were calculated by first ensemble averaging the BP beats, referenced by the R-peak onset [35]. The peaks and valleys of each beat correspond to systolic (SBP) and diastolic (DBP) blood pressures, respectively. Pulse pressure (PP) was calculated by measuring the difference between SBP and DBP.

### Statistical analysis

Stress reactivity was evaluated by comparing the first minute of stress with a one-minute rest period that started five minutes prior to stress-onset. Only one minute was evaluated for both cohorts, because the stress duration in the healthy cohort was one minute. To affirm whether significant hemodynamic changes occur from the first minute of stress to warrant its use for the aging group with CAD (instead of the mean for the entire stress period), a supplementary analysis was performed using the vital measures HR, SBP, DBP, PP to evaluate changes from baseline to the first minute of stress and mean overall stress. Statistical significance was tested with a one-way analysis of variance (ANOVA) for normally distributed data, or Kruskal-Wallis for non-normal data. Later, follow-up parametric or non-parametric multiple comparisons tests based on Tukey-Kramer honest significant difference criterion were performed to understand which intervals significantly differ from each other. Stress reactivity was measured by subtracting stress values from rest values. A paired t-test was used to compare stress versus rest. Cohen’s distance was calculated using the mean of two states for each group, and converted to effect size [41]. A two-sample t-test for unequal variances was used to compare the stress-to-rest ratios between the cohorts. The evaluation for covariate effects was limited due to the small sample sizes. Nonetheless, because of the potential role of sociodemographic and health factors, an exploratory analysis was performed to evaluate for group differences by risk factor. Linear regression models were performed to adjust for age, sex, beta-blocker usage, ACE inhibitor usage, and antidepressant usage when calculating group-level differences. 95% confidence intervals for physiological markers were calculated from the mean and standard deviation of differences between stress and rest states [42]. The proportion of subjects whose physiological values increased or decreased with stress was also tabulated as a secondary method of evaluating stress reactivity; for this, Chi-square or Fisher’s exact test was used to detect group differences, where appropriate [43]. For all statistical analyses, p-values ≤ 0.05 were considered significant. All statistical analyses were carried out using MATLAB Statistics and Machine Learning Toolbox.

## Results

In the healthy group, 80% were male and the mean (SD) age was 25.4 (4.4) years. In the aging group, 68% were male and the mean (SD) age was 64.8 (5.9) years. Table 2 presents the mean, standard error of the mean, percent changes, confidence intervals, p-values, effect sizes of the physiological markers extracted from ECG, ICG, and BP signals for each study, for rest and stress states. Due to device malfunction for one subject, BP signals were collected from 24 patients. As the primary outcomes, PEP decreased significantly for both groups, while HF HRV decreased significantly only in the aging group.

**Table 2:** Data for both groups: Rest, stress and mean of differences.

	Healthy Group (n = 25)						Aging Group (n = 25¶)					
	Rest	Stress	S-R	95% CI	P	r	Rest	Stress	S-R	95% CI	P	r
**HR [bpm]**	92.9 (2.5)	101.3 (3.4)	8.4*	(3, 13.7)	<0.01	0.28	62 (1.8)	69.4 (2.5)	7.4*	(4.1, 10.6)	<0.001	0.32
**PEP** **[ms]**	109.3 (3.9)	99.7 (4.3)	-9.6*	(-14.2, -5.1)	<0.001	0.23	80.9 (4.3)	74.5 (3.8)	-6.4*	(-10.9, -2)	<0.01	0.16
**HF HRV [log-ms**^**2**^**]**	2 (0.1)	1.9 (0.1)	-0.1	(-0.4, 0.2)	0.71	0.1	2.6 (0.2)	2.3 (0.2)	-0.4*	(-0.6, -0.1)	0.01	0.21
**SBP [mmHg]**	117.4 (3.5)	128.5 (3.9)	11*	(8.3, 13.7)	<0.001	0.29	130.4 (3.7)	147.4 (6.1)	17*	(10.7, 23.2)	<0.001	0.22
**DBP [mmHg]**	75.9 (1.7)	81.6 (1.8)	5.7*	(4.3, 7.1)	<0.001	0.31	74.8 (2)	77.8 (2.7)	3	(-0.2, 6.2)	0.09	0.08
**PP [mmHg]**	41.6 (2.1)	46.9 (2.4)	5.3*	(3.5, 7.2)	<0.001	0.23	55.6 (2.9)	69.6 (4.4)	14*	(9, 19)	<0.001	0.3
**std HR [bpm]**	5.6 (0.9)	5.9 (0.5)	0.2	(-1.4, 1.9)	0.79	0.49	4 (0.5)	3.8 (0.5)	-0.1	(-1.1, 0.9)	0.82	0.02

Values represent mean (SEM).

* Denotes change in feature from rest to stress is significant, P≤0.05.

^¶^ Denotes n = 24 for SBP, DBP, PP.

S-R: Mean of differences between states, calculated by stress-rest for each subject for the corresponding feature.

95% CI: 95% Confidence Interval (Lower bound, Upper bound).

P: P-value.

r: Effect size.

The healthy group showed 50% higher PEP decrease (9.6ms) than the aging group (6.4ms) during mental stress. In total, five physiological markers (HR, PEP, SBP, DBP, PP) were found to be significantly different between stress and rest for the healthy cohort (n = 25). Five physiological markers (HR, PEP, SBP, PP, HF HRV) were also found to be significantly different between stress and rest for the aging cohort (n = 24 for BP levels, n = 25 for other markers).

No statistically significant or large effect size difference (*r*<0.4) in stress reactivity was found between groups, regardless of adjustment for age, sex, beta-blocker usage, ACE inhibitor usage, or antidepressant usage. We also explored the possibility of gender-related bias in healthy population: within the young group, there is no statistically significant or meaningful effect size difference between the stress responses between young men and young women in any of the measured parameters. Table 3 compares the directionality of physiological changes from rest to stress. For the healthy cohort, 72%, 88%, and 56% of the subjects showed increased HR, decreased PEP, and decreased HF HRV, respectively. The corresponding percentages were all 80% for the aging cohort. The proportion of subjects with a decrease in HF HRV was 24% less in the healthy cohort compared to the aging cohort (p = 0.08).

**Table 3:** Comparison of ANS activity biomarkers.

ANS Activity Indicators	Healthy (n = 25)	Aging (n = 25)	Difference
**HR increase**	72%	80%	8%
**PEP decrease**	88%	80%	8%
**HF HRV decrease**	56%	80%	24%

Values represent the percentages of each population (n = 25 healthy subjects, n = 25 aging subjects).

**Fig 3:**
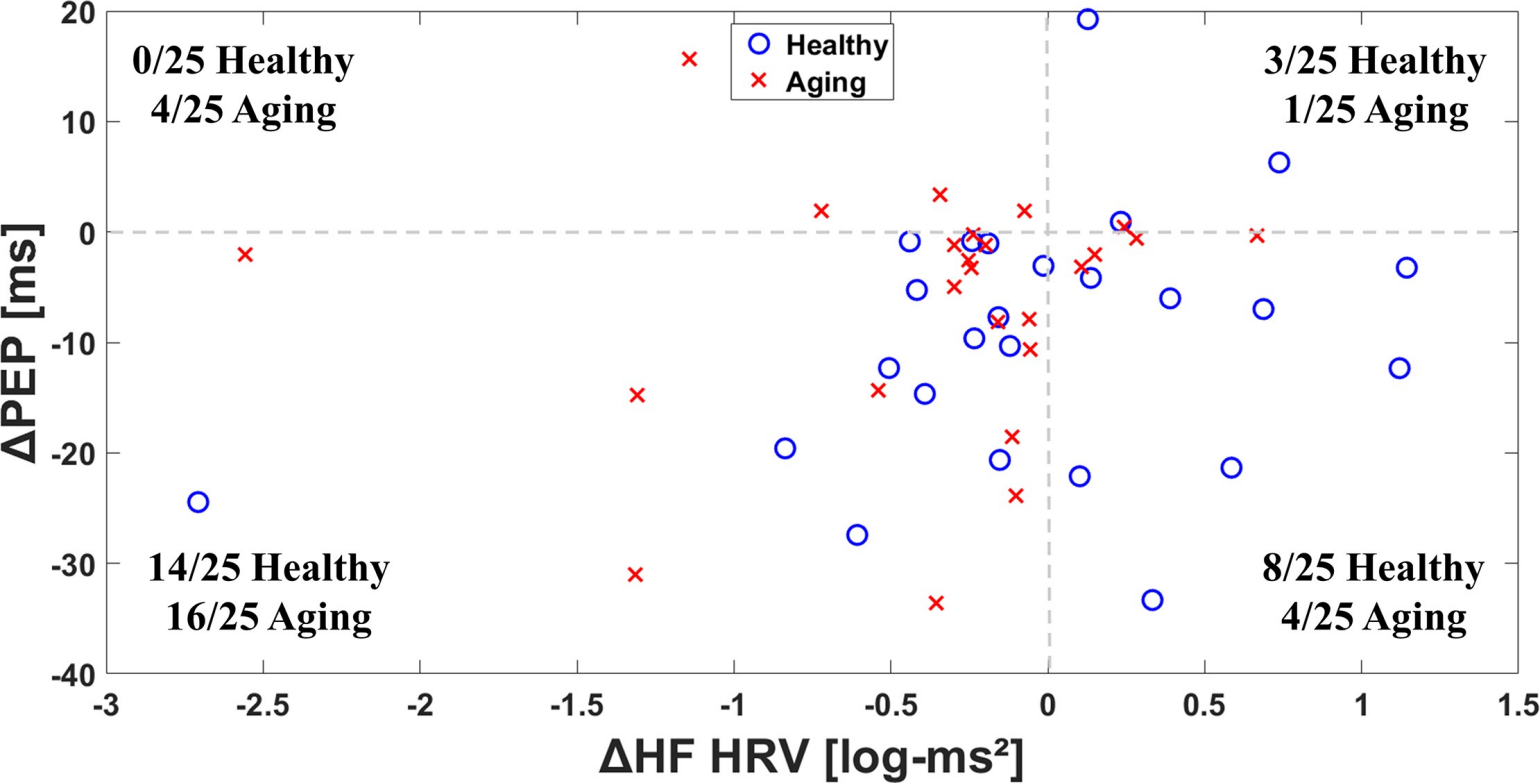
Changes in HR HRV and PEP responses, shown as ΔHF HRV vs. ΔPEP. Values show difference between stress and rest, as stress-rest. Each quadrant shows the number of aging and young/healthy subjects.

Fig 3 summarizes the distributions of the stress-minus-rest values for HF HRV and PEP. Most subjects in both groups showed decreases in both values, consistent with increase in SNS and decrease in PNS during stress. No statistically significant differences were found between groups in individual quadrants.

## Discussion

This study represents the first attempt, to our knowledge, of assessing cardiac SNS and PNS mental stress reactivity in healthy versus aging subjects with CAD; to our surprise, we found a large proportion (44%) of subjects in the healthy group had paradoxical increases in HF HRV, a marker of PNS activity, and a 20% of subjects in the aging group demonstrated decreases in PEP, a marker of SNS reactivity. While the differences between groups were not statistically significant, the nature of this study was exploratory. Overall, this study warrants further evaluation of physiologic stress reactivity as it relates to aging and disease, and supports the notion that, in many cases, the expected increase in SNS and decrease in PNS with mental stress challenge do not occur. This may be related to pathological reasons, like myocardial ischemia (which would increase PEP) or reflexive PNS activation (which would decrease HF HRV).

The increased PNS activity in response to mental stress that was largely noted in the healthy group is potentially adaptive [44]. It may be a compensatory autonomic reflex to the sympathetic stress response, as a way to buffer against the pathophysiologic effects of sympathetic activation. Such mechanisms may perhaps be disrupted in subjects with CAD and explain the lower proportion of CAD subjects who demonstrated an increase in PNS: cardiac PNS activity was previously studied to decrease with age [45], and it is known that there are histological and functional reductions in sinus node during aging [46–48]. Aging populations are more likely to have sinus node dysfunction, which decreases their PNS regulation ability through sinus node [49], as they are more susceptible to stress effects. Young people, on the other hand, would have better PNS regulation through sinus node as there is not as much deterioration. The increased PNS activity in some young healthy adults might be due to this respiratory-activated PNS activity. Young individuals may also have more intact baroreflex mechanisms, which activate the PNS in response to increased blood pressure. This may have occurred since the younger group was observed to have a hypertensive response during stress. Aging group with CAD experienced significant decrease in HF HRV likely because the sinus node activity or baroreflex mechanisms were impaired.

Although mean PEP decreased, a small percentage of each group showed increased PEP with stress. In the aging cohort who had decreased HF HRV to indicate PNS withdrawal, prolonged PEP may be a consequence of impaired mechanical contraction secondary to ischemia, transient conduction disease, or other causes [50–52]. Impaired baseline left ventricular function was also noted in one of the subjects in the aging group with increased PEP. It may also suggest that the arithmetic challenge did not induce significant mental stress. Overall, these paradoxical PEP increases during mental stress warrants further investigation and may suggest increased risk.

The reasons for potential differences cannot be attributed to any single factor. The aging group had high prevalence of hypertension (68%), dyslipidemia (80%) and diabetes (40%); 48% of this cohort also had history with smoking. These conditions, in addition to CAD, may have contributed to the decrease in HF HRV with mental stress as well [22, 53]. Medications may also explain a difference in HF HRV between groups, although the aging group was asked to withhold beta-blockers in the morning before the procedure, which are one of the main potential confounders.

Differences in baseline blood pressure and its stress reactivity were noted, with higher values in the aging group. Such differences are consistent with prior literature [54–56]. The group difference in pulse pressure are the most notable; the mean change from stress to rest (14 mmHg) in the aging group was nearly three-fold higher than the mean change in healthy group (5 mmHg). In multivariable models, the differences were not significant after adjustment for age and sex, however. Short-term changes in blood pressure are mainly controlled by the baroreflex, which is one of the major determinants of SNS activity [57]. Aging subjects have higher blood pressure (SBP, DBP, PP) during baseline and mental stress, compared to the young healthy group, and their stress responsivity in terms of SBP and PP is higher, consistent with previous studies [58]. The three-fold difference in PP between groups signal that age-related changes in baroreceptors increase sympathetic responsivity to mental stress.

This study is subject to limitations. We compared stress reactivity from two independently conducted studies in an exploratory analysis. We felt this was justified because both utilized arithmetic stressor tasks, and the majority of the findings are in agreement with the literature and credible biological concepts [11–15, 18–21]. We restricted our analysis to the first minute of stress only, and this was justified by findings that stress reactivity was consistently higher in the first minute compared to the entire stress period (see S1 Table). S1 Table lists the p-values and results from multiple comparisons for the three intervals, indicating that the hemodynamic changes occur significantly between baseline and the first minute of stress: data from overall stress interval are significantly higher from baseline only for PP case (p = 0.0025), while data from the first minute of stress are significantly higher than the baseline for HR (p = 0.0208), SBP (p = 0.0095), PP (p = 0.0025). We have also found in previous analyses that different stressors may yield similar results in the brain, suggesting consistent neurobiology across stressor types [59]. Furthermore, as per Table 2, HR and SBP responses between groups were similar between studies, which suggest a similar overall net stress responsivity with regards to some summative measures. On the other hand, differences in DBP reactivity can be explained by differences in vascular stiffness that occur as a part of aging [60]. Confounding from CAD may have also played a role, although we found that within the aging group, CAD burden (Gensini score) did not associate with stress reactivity (see S2 Table), and therefore unlikely to be a confounder. Nonetheless, the results could be strengthened if the study included an older group without CAD. Data on such subjects have been published elsewhere; a group of older participants with mean (SD) age of 67 (1) underwent Trier Social Stress Test and demonstrated a similar heart rate response [61]. In another study of CAD patients versus healthy controls, CAD status did not associate with changes in heart rate reactivity to stress [62].

Although our older group has CAD and other comorbidities, as well as medication exposures, our sample size does not adequately allow comparisons between groups or within groups. Therefore, we describe the differences between groups due to aging, which is nonspecific, but nonetheless summarizes the large number of comorbidities that are usually associated with the aging process (such as hypertension [63]). Despite the small sample size, working between two extremities in age/health status increases the likelihood of capturing the group differences. Another limitation is that all women in the aging group were post-menopausal, and as such we could not evaluate the effects of this specifically. Menopause is an important aspect of aging in women, and may have had an independent effect on stress reactivity in the women of the aging group response (i.e. increase in stress response as observed by [61]). We were able to only report minimal HRV metrics because of the minimum ECG signal length for the spectral analysis of HRV. Frequency-domain HRV analysis requires approximately one minute of ECG recording for assessing high frequency [62] HRV (>6 cycles), and two minutes of recording for assessing low frequency (LF) HRV [23]. HF HRV reflects changes in the PNS activity, whereas LF HRV is influenced by both SNS and PNS activity. The ratio of both powers, LF/HF HRV, is widely adopted in the literature to be an indicator of sympathetic tone, however multiple studies proved its inconsistency to reflect sympathetic control on the heart [23–25, 27]. As we were primarily interested in PNS influences during the one-minute mental stress testing in our study, we only reported HF HRV results.

Our findings underscore the need for multiple physiologic monitoring methods to assess the effects of acute mental stress on cardiovascular physiology. Although ECG is commonly measured, ICG is less commonly employed. For ambulatory studies, this can be more difficult, although new devices allow for real-time monitoring of stressful situations. The VU-AMS monitor used in this study can potentially be combined with ecological stress monitoring to comprehensively assess psychophysiological reactivity in everyday circumstances [64]. HF HRV is a standardized biomarker to assess PNS activity [23], however SNS activity quantification largely differs in clinical studies. Non-invasive techniques widely acquired to quantify SNS activity are ECG-based HRV measures and electrodermal activity (EDA) [23, 65]. Frequency-domain based HRV measures (LF HRV) are convenient to measure, however there are disagreements in the scientific community regarding its merit in quantifying SNS activity in particular, as LF HRV is influenced by both SNS and PNS activity [23]. PEP is an underused measure in clinical studies due to the requirement for impedance cardiography, however its use to reflect the SNS activity was studied by many groups: a comparison of PEP and LF/HF HRV showed that PEP outperforms LF/HF for conditions that are known to increase sympathetic activity, or LF/HF is specifically shown unable to reflect changes in SNS activity, such as mental stress [66], exercise [67], and beta-adrenergic/cholinergic blockade [26, 68]. Additionally, as a validation of PEP with EDA, studies have shown PEP to classify electrodermally labile and stabile subjects during rest and during easy and hard arithmetic mental stressors. Also, both indices (EDA and PEP) change significantly during mental arithmetic [69]. PEP and HF HRV are convenient biomarkers for this purpose as they are non-invasive, non-obtrusive, and can be continuously assessed. Newer markers of SNS activity, such as periodic repolarization dynamics, which only requires ECG [70, 71], may reduce the need for ICG when measuring cardiac SNS activity, although its role in ambulatory settings warrants further investigation.

In summary, we performed an analysis of SNS and PNS responses to acute mental stress in healthy and aging subjects. PEP decreases with stress regardless of health and age status, as anticipated. Contrary to what we expected, we found that many (44%) healthy subjects did not experience vagal withdrawal, and 20% of aging subjects demonstrated paradoxical increases in PEP. The differences between groups are not statistically significant as our sample size is limited, p-values are often misused and lead to publication bias [72]. The fundamental limitation of our study is small sample size, but the utility of the study is the preliminary data that it provides for the larger investigation that is based both on the data, and the fundamental aspects in biology. The results highlight possible limitations of these measures in psychophysiology studies, as well as the need to include multiple complementary autonomic measurement modalities. More research is needed to verify these findings and understand their potential implications as they relate to emotionally-triggered adverse outcomes, especially in the aging cohort.

## Supporting information

S1 CONSORT Checklist Consolidated Standards of Reporting Trials (CONSORT) checklist of the manuscript. (DOC)

S1 Data Lists the physiological outcomes for each young, heathy and aging subject with CAD, each are in separate sheets. (XLSX)

S1 Table Statistical analyses on the CAD group’s baseline, first minute of stress, and overall stress on HR, SBP, DBP, PP. Baseline, first minute of stress, overall stress values represent mean (SD). P-values were calculated from ANOVA (for normal data) or Kruskal-Wallis (for non-normal data) on the three intervals (baseline, first minute of stress, overall stress), and results from follow-up multiple comparisons comparing the intervals were reported.
(PDF)

S2 Table R-squared values and 95% CI for stress reactivity and Gensini scores. R-squared values and 95% CI (lower bound, upper bound) values from the linear models derived from the change in each physiological measure and the corresponding Gensini score. The changes were assessed as the difference between stress and rest values for each physiological measure.
(PDF)

S1 Biofeedback Study Protocol Effects of a behavioral intervention using biofeedback on myocardial blood flow changes during mental stress in patients with coronary artery disease. Protocol document provides detailed description of each intervention applied to patients with CAD.
(PDF)

## Abbreviations

ACE: angiotensin converting enzyme
ANS: autonomic nervous system
BP: blood pressure
CABG: coronary artery bypass grift
CAD: coronary artery disease
CI: confidence interval
DBP: diastolic blood pressure
ECG: electrocardiography
EF: ejection fraction
FIR: finite impulse response
HF HRV: high-frequency heart rate variability
HR: heart rate
ICG: impedance cardiography
LF HRV: low-frequency heart rate variability
MBF: myocardial blood flow
PEP: pre-ejection period
PNS: parasympathetic nervous system
PP: pulse pressure
PSD: power spectral density
PTCA: percutaneous transluminal coronary angioplasty
SBP: systolic blood pressure
SEM: standard error of the mean
SNS: sympathetic nervous system
