# Supplementary material for: Comparison of autonomic stress reactivity in young healthy versus aging subjects with heart disease
Source: PLoS One. 2019 May 8;14(5):e0216278. doi: 10.1371/journal.pone.0216278 (PMC6505888; doi:10.1371/journal.pone.0216278)
Supplement: S1 Table — Baseline, first minute of stress, overall stress values represent mean (SD). P-values were calculated from ANOVA (for normal data) or Kruskal-Wallis (for non-normal data) on the three intervals (baseline, first minute of stress, overall stress), and results from follow-up multiple comparisons comparing the intervals were reported. (PDF) [file pone.0216278.s003.pdf]

**S1 Table. Statistical analyses on the CAD group's baseline, first minute of stress, and overall stress on HR, SBP, DBP, PP.**

| Stress Reactivity Measure | Baseline | Stress (First Minute) | Stress (Overall) | P-value | Multiple Comparisons Result |
|---|---|---|---|---|---|
| **HR [bpm]** | 61 (10) | 70 (13) | 69 (12) | 0.0208 | First minute of stress is higher than baseline. |
| **SBP [mmHg]** | 129 (18) | 149 (29) | 145 (23) | 0.0095 | First minute of stress is higher than baseline. |
| **DBP [mmHg]** | 74 (10) | 78 (13) | 76 (11) | >0.05 | No difference between intervals. |
| **PP [mmHg]** | 55 (14) | 72 (20) | 69 (16) | 0.0025 | Both first minute and overall stress values are higher than baseline. |

Baseline, first minute of stress, overall stress values represent mean (SD). P-values were calculated from ANOVA (for normal data) or Kruskal-Wallis (for non-normal data) on the three intervals (baseline, first minute of stress, overall stress), and results from follow-up multiple comparisons comparing the intervals were reported.
