# Supplementary material for: Comparison of autonomic stress reactivity in young healthy versus aging subjects with heart disease
Source: PLoS One. 2019 May 8;14(5):e0216278. doi: 10.1371/journal.pone.0216278 (PMC6505888; doi:10.1371/journal.pone.0216278)
Supplement: S2 Table — R-squared values and 95% CI (lower bound, upper bound) values from the linear models derived from the change in each physiological measure and the corresponding Gensini score. The changes were assessed as the difference between stress and rest values for each physiological measure. (PDF) [file pone.0216278.s004.pdf]

S2 Table. R-squared values and 95% CI for stress reactivity and Gensini scores.

Stress Reactivity Measure	R-squared Value	95% CI
**HR**	2.850e-04	(-0.0743, 0.0688)
**Std HR**	0.153	(-0.0008, 0.0403)
**PEP**	2.470e-04	(-0.1024, 0.0954)
**HF HRV**	6.590e-04	(-0.0062, 0.0055)
**SBP**	0.048	(-0.0706, 0.2080)
**DBP**	0.177	(0.0014, 0.1373)
**PP**	6.15e-06	(-0.1159, 0.1147)

R-squared values and 95% CI (lower bound, upper bound) values from the linear models derived from the change in each physiological measure and the corresponding Gensini score. The changes were assessed as the difference between stress and rest values for each physiological measure.
