# Supplementary material for: Comparison of autonomic stress reactivity in young healthy versus aging subjects with heart disease
Source: PLoS One. 2019 May 8;14(5):e0216278. doi: 10.1371/journal.pone.0216278 (PMC6505888; doi:10.1371/journal.pone.0216278)
Supplement: S1 Biofeedback Study Protocol — Protocol document provides detailed description of each intervention applied to patients with CAD. (PDF) [file pone.0216278.s005.pdf]

Effects of a Behavioral Intervention using Biofeedback on Myocardial Blood Flow Changes during Mental Stress in Patients with Coronary Artery Disease (MIBS: Mental Stress Ischemia: Biofeedback Study)

Wilmot K, Garcia E, Bremner D, Shah AJ, Hummadah, M.,Nye J, Raggi P, Kutner M, Pearce B, Al-Mheid I, Murrah N, Shallenberger L, Vaccarino V, Quyyumi AA

Precis/Abstract:

Psychological stress has been implicated in the pathophysiology of coronary artery disease (CAD). Myocardial ischemia can be induced by a standardized psychological challenge in some patients with CAD. This mental-stress induced myocardial ischemia (MSI) may only be provoked by a psychological stimulus rather than exercise stress. Although it is a predictor of poor prognosis, the exact mechanisms underlying MSI are unknown. One primary proposed mechanism is reduced coronary perfusion secondary to microvascular constriction that may be a result of endothelial dysfunction. Thus, assessing myocardial blood flow during mental stress would be valuable in evaluating the coronary vascular responses to psychological stress. PET measurements of myocardial blood flow (MBF) using Rb^{82} are well-established techniques. Peripheral arterial tonometry (PAT) measures digital microvascular tone and vasomotor changes during stress. Changes in the fingertip may mirror coronary vascular responses and help to gain insight on the microvascular responses during stress. Heart rate variability (HRV) biofeedback is known to improve both psychological and physiologic well-being. Previous studies have found HRV biofeedback techniques to improve mood, reduce cortisol, increase baroreflex gain, and reduce blood pressure.[1-3] Whether the beneficial effects of biobehavioral therapy on autonomic tone and vascular function will improve coronary and peripheral blood flow during mental stress has not been previously evaluated and is the aim of our proposal.

In this pilot study, we will enroll 24 patients with CAD to be randomized 1:1 to HRV biofeedback therapy or waitlist control. Patients with known mental stress ischemia based on our previous study, "Mental Stress Ischemia: Genetic Mechanisms and Prognosis," (MIPS, IRB 35540) will be recruited preferentially. All patients will undergo myocardial flow/perfusion imaging with Rb^{82} PET imaging at rest and after a standardized arithmetic mental stress test. They will then undergo repeat testing after 8 weeks. At 16 weeks, they will also undergo a limited examination without myocardial perfusion imaging. The intervention group will receive biofeedback after enrollment, and the wait-list control group will receive the intervention between the 2nd (8 week) and 3rd (16 week) study visit (without imaging). We will also evaluate peripheral vascular function including (a) endothelial function as flow-mediated vasodilation, (b) arterial stiffness, and (c) PAT. Finally, autonomic function will be measured, including high frequency (HF) HRV (parasympathetic), pre-ejection period (sympathetic), and plasma catecholamines (sympathetic).

The aims of the study are to assess the effects of HRV biofeedback (versus usual care) on 1) global and regional MBF; 2) peripheral vascular function; and 3) autonomic changes during mental stress.

As a secondary analysis, the effects of HRV biofeedback on coronary flow reserve (using pharmacologic stress) will be performed on a subset of ½ patients.

Introduction and Background

There is growing recognition of the detrimental effects of psychological and emotional stress on coronary heart disease (CHD) incidence and mortality. Psychological stress has been implicated in the pathophysiology of CHD by triggering acute coronary events, exacerbating CHD risk factors, influencing development of atherosclerosis, and impairing the recovery, prognosis and quality of life of patients after an acute coronary syndrome.[4] In up to half of patients with CHD, myocardial ischemia can be induced by a standardized psychological challenge. This mental-stress induced myocardial ischemia (MSI) is analogous to exercise stress ischemia, except that the stimulus is psychological rather than physical or pharmacological stress.[5,6] It is typically painless, occurs at lower levels of oxygen demand than exercise ischemia, is generally not related to severity of coronary artery disease (CAD), and is a predictor of poor prognosis.[7-9] However, the exact mechanisms underlying MSI and its adverse prognostic significance are unknown. One proposed mechanism is reduced coronary perfusion secondary to impaired vasodilation of the coronary circulation during mental stress that may primarily be an abnormality of the coronary microcirculation.[10,11] Patients with CAD who develop MSI appear to be especially vulnerable to psychological stress and thus represent an ideal group to study the effects of intervention on myocardial perfusion changes during psychological stress.

Peripheral or pulsatile arterial tonometry (PAT) is a vascular test developed to evaluate digital microvascular vasodilator function in the fingertip. PAT reactive hyperemia predicts short and long term risk of cardiovascular events.[12] We have shown that patients with CAD who do not develop MSI during laboratory psychological testing have significantly greater digital vasodilation, measured using PAT, than patients with MSI.[13-15]

Evaluating myocardial perfusion is receiving growing attention in the assessment of the pathophysiological processes underlying myocardial response to psychological stress. PET measurements of myocardial blood flow (MBF) and myocardial flow reserve (MFR) using Rb^{82} are well-established techniques, both in research protocols and clinically. Arrighi *et al.* reported blunted augmentation of MBF, as measured by [^{13}N] ammonia PET, during mental stress in regions without significant epicardial stenosis, suggesting a prominent role for microvascular dysfunction.[16] Thus PET is ideal for measuring mental stress-induced flow impairment due to either epicardial and/or microvascular disease.

Even though there is evidence of the significance of psychological stress on CHD pathophysiology,[17] there is a dearth of effective behavioral interventions to mitigate the adverse effects of stress in CHD patients. Earlier studies of behavioral or psychological interventions have reported beneficial effects. In particular, stress management techniques reduce depressive symptoms, improve endothelial function measured as flow-mediated dilation, improve left ventricular ejection fraction responses to stress, and improve risk of cardiac events.[18-20] More recently, Linden *et al.* performed a meta-analysis on the effect of psychological treatments in

cardiac patients.[21] Mortality benefits were reported with the addition of psychological treatments to usual care, especially when started early after an acute event. However, it was unclear as to which intervention was most likely to benefit.[21]

In this pilot study in patients with CAD, we will use Rb^{82} PET to measure myocardial blood flow (MBF) before and after six weeks of heart rate variability (HRV) biofeedback. HRV biofeedback combines deep breathing, mindfulness, and cognitive therapy, and has been found to improve depressive symptoms and autonomic function, and has a high likelihood of success in this patient population.[22-24]

Objectives

Primary Objective

Study the effects of a 6-week behavioral intervention using biofeedback (versus usual care) on global MBF and MFR during mental stress. **We hypothesize that patients who undergo a biofeedback intervention (vs. wait-listed controls) will have an increase in global and regional myocardial perfusion during mental stress (measured as Rb^{82}PET MBF).**

Secondary objectives

1. Study effects of biofeedback therapy on myocardial perfusion. **We hypothesize that the number of ischemic regions will decrease.**
2. Study the effects of biofeedback on peripheral vascular changes and biomarkers of sympathetic nervous system activation during mental stress. **We hypothesize that compared to controls, patients who undergo biofeedback (vs. usual care) will have less vasoconstriction (higher PAT ratio), lower resting catecholamine levels (norepinephrine, epinephrine and dopamine, pre-ejection period), and a reduced catecholamine stress response post-intervention vs. pre-intervention.**
3. Assess the relationship between stress-induced digital blood flow changes (PAT ratio), catecholamine increases, and changes in MBF before and after biofeedback intervention. **We hypothesize that compared to controls, patients with biofeedback therapy will have greater improvement in PAT ratio and lowest increases in catecholamines compared with baseline (pre-intervention).**
4. Assess the relationship between stress-induced endothelium dependent flow-mediated dilation (FMD) and arterial compliance (augmentation index and pulse wave velocity) and changes in MBF before and after biofeedback intervention. **We hypothesize that compared to controls, patients with biofeedback therapy will have greater improvement in FMD and arterial compliance.**

Figure 1: PET Mental Stress and HRV Biofeedback Protocol

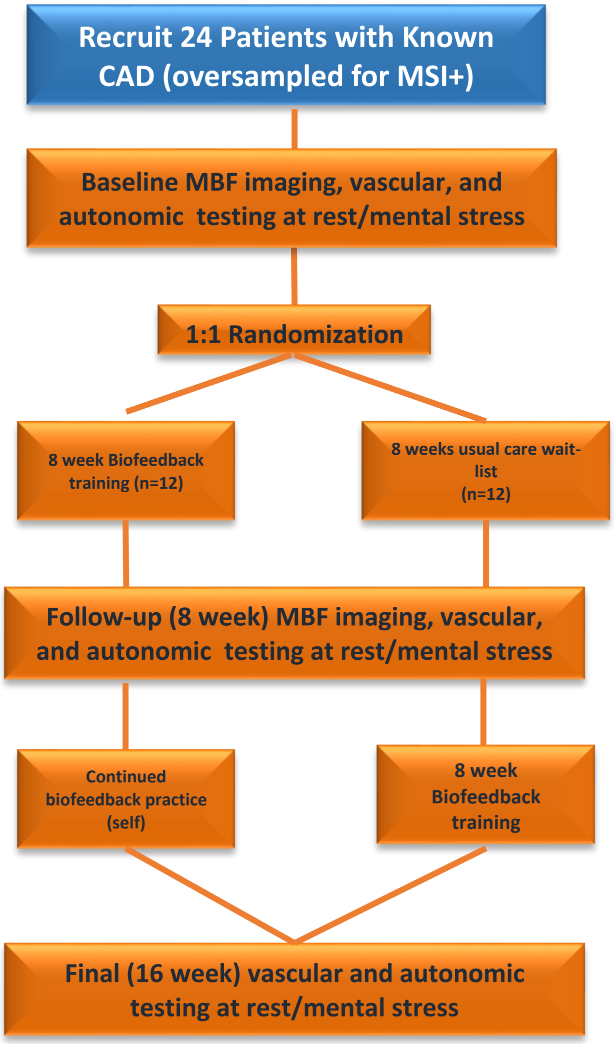

Study Design and Methods

Overall Study Design

This pilot-study (Figure 1) will recruit 24 patients from the Mental Stress Ischemia Mechanisms and Prognosis Study (MIPS), a longitudinal cohort study which enrolled 695 patients between July 2009 and July 2014 with a diagnosis of stable CAD from Emory University-affiliated hospitals or clinics. Each patient completed baseline myocardial SPECT imaging, as well as psychosocial, hemodynamic and microvascular assessments, and blood was obtained for inflammatory, neurohormonal, and immunological biomarkers prior, during and after mental stress and conventional (exercise or pharmacological) stress. These patients have been followed on regular intervals with in-person clinic visits every year and by telephone every 6 months.

The patients will be randomized 1:1 to HRV biofeedback intervention for 8 weeks or wait-list control. Each group will undergo a rest/stress Rb^{82} PET imaging protocol (detailed below) similar to regular clinical imaging except that stress will be performed with a standardized arithmetic mental stress test rather than pharmacological stress. We will hold beta-blockers for 48 hours and calcium channel blockers and nitrates on the day of the testing. It is standard protocol to hold anti-ischemic medications, including beta-blockers, ace-inhibitors, calcium channel blockers and nitrates, before exercise stress imaging studies,[25] and this is the protocol followed by other nuclear cardiology studies.[26,27]

Figure 2: Protocol

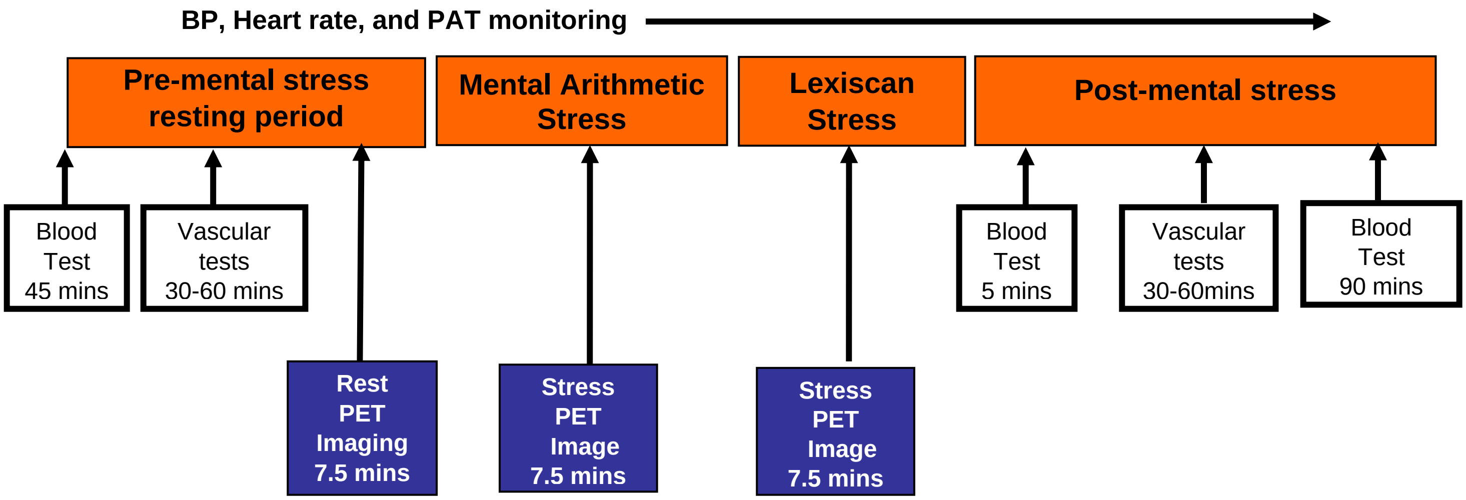

The full protocol is detailed in Figure 2.

Before Mental Stress Testing: Patients will be studied in the morning after an overnight fast. After consent, patients will have 20 ml blood drawn for measurements of catecholamine levels, and inflammatory markers. They will be outfitted with a combined ECG/ICG monitor, the VU AMS holter, which is a 5-lead recorder that measures cardiac impedance and ECG for the autonomic measures. Additionally, they will measure a fitness bracelet, the Empatica E4, to measure photoplethysmography and actigraphy. In a quiet temperature controlled Vascular Laboratory, after at least 15 minute rest period, we will measure arterial compliance (augmentation index and pulse wave velocity) using the SphygmaCor device. Immediately following this, patients will be fitted with the tonometry device (PAT, Itamar Inc), and we will measure endothelial function by brachial artery reactivity testing and simultaneously also measure PAT reactive hyperemia index. Once this test is completed within 15 to 20 minutes, patients will be moved to the PET laboratory for a 30-minute rest period before the mental stress test. During this period, heart rate and blood pressure will be measured at every 5 minutes (automatic oscillometric device (Datascope Accutorr) and a continuous recording of microvascular finger blood flow using the PAT device will continue. They will continue to wear

the VU AMS device and Empatica bracelet, and also undergo additional 12-lead ECG monitoring during the stress to evaluating for arrhythmia/ST changes. The resting study Rubidium^{82} infusion and PET image acquisition will be obtained approximately 7.5 mins prior to mental stress. 30-60 mCi of Rubidium^{82} (based on patient body weight) will be injected in total.

Mental Stress Protocol

Mental stress will consist of a 3-min math serial subtraction paradigm previously described by Soufer et al.[28] The subjects will be asked to serially subtract 7 from number specified (i.e., 986) by the researcher; for patients who have difficulty with this task, easier serial subtraction will be provided for (e.g., by 4, 3, or 2). Throughout the task, to increase stressfulness and titrate difficulty, the patient will be prompted for faster performance and the starting number from which they were subtracting will be periodically changed (e.g., if the starting number was 986, it may be changed to 549 after performing a number of successful serial subtractions). Finally, to add an evaluation component, patients will be given negative feedback during the test. Blood pressure and heart rate will be recorded at 1-minute intervals. Subjective stress levels will be evaluated by the use of a visual analog scale; this will be repeated at the end of the stress test. At 60 seconds into the mental stress task, 30-60 mCi of Rubidium^{82} (based on patient body weight) will be injected; images will be acquired as described below.

Post mental stress testing: Heart rate and blood pressure will be measured every minute for the first 5 minutes and at 5 minute intervals after the end of the mental test. Microvascular blood flow will also be continuously measured by PAT. Five minutes after the end of the test, 20 ml of blood will be drawn for catecholamine levels. Fifteen minutes after the mental stress test, arterial compliance measures will be repeated. Thirty minutes after the end of the test, brachial reactivity and simultaneous PAT reactive hyperemia will be repeated and the PAT device disconnected. Ninety minutes after stress, another 20 ml sample of blood will be drawn for future studies involving post-stress inflammatory measures, determined at a later date.

Biofeedback Intervention Protocol

The biofeedback intervention will be directed by Dr. Ginsberg, a clinical neuropsychologist at the Medical University of South Carolina (he will be hired as a consultant), and Dr. Shah, who is trained in biofeedback therapy. A local biofeedback trainer with significant experience and certification will be hired for the intervention. Additionally, an experienced off-site HRVB trainer will be contracted to provide phone training for ½ of the sessions. HRVB training is conducted in an interactive manner using a computerized system to monitor and display the individuals' HRV patterns in real time while their heart rate is recorded. Visual HRV feedback (either a quantitative display or animated challenge games) is provided as participants practice attention focusing, resonant frequency breathing, imagery, and the induction of positive emotion. The resulting changes in HRV patterns are readily observed by the subject. The visual feedback enables associations to be formed between these techniques and HRV patterns indicative of higher vagal parasympathetic output. Before HRVB training, the biofeedback trainer discusses individual issues and the training goals of the participant. The physiological connection between resonant frequency breathing and heart rate is introduced. This is reinforced with corrective

breathing instruction and help finding the resonant frequency (~6 respirations/min). Dual monitors also display the HRV power spectrum in real-time so that the participant can visualize the impact of proper breathing on the coherence peak at 0.10 Hz. Positive emotion and interactive visual images are then introduced. This interactive process allows the participant to customize a sustainable system, with occasional shifts back to the power spectrum screen to observe the 0.10 Hz HRV peak. During the recording period after coaching, subjects view a static nature scene on the computer while their heart rate is measured by ECG.

Adherence Assessment

The biofeedback coach will instruct participants in both groups to practice their training at home a minimum of 10 minutes a day, and participants will be given a log to record their daily home practice. Home practice self-report data will be incorporated into the analysis to examine the potential dose-response effects of home practice. Information will be solicited in a questionnaire at the start of each lab regarding the factors that contributed to or detracted from home practice, for inclusion in the analysis. One goal of this intervention is to spontaneously and consistently achieve HRV coherence on demand outside a clinical environment. Therefore, participants will be provided with a portable colorimetric finger PPG hand held personal stress reliever; dimensions similar to a cell phone; weight: 2.2 ounces for home practice and use between weekly HRVB training sessions. Home HRVB practice period data from the device can be compared to self-report and further analyzed with regard to the outcomes.

Personal HRVB coaching will be provided by HRVB trainer/mentors. These individuals are trained to teach the practical application of HRVB. Three individual mentoring sessions per participant will be provided via telephone or online via Skype to supplement acquisition of core HRVB skills through home practice. Through this program, the methods of achieving and sustaining HRV coherence are reinforced.

PET Imaging Protocol

All studies will be acquired using a Siemens Biograph-40 3D PET/CT scanner (Knoxville, TN) comprised of a lutetiun oxyorthosilicate (LSO) block detector ring of 162 mm field-of-view operating in 3D mode.

Images are acquired as follows: a scout is done to define scan range. A CT attenuation scan is performed with the lowest pitch allowed by the scanner, covering the region of interest in 14 seconds during shallow breathing. Two rubidium infusions of 1295 – 2220 MBq (35-60 mCi) are performed. A 7.5-minute rest emission scan in list mode is started immediately after the beginning of rubidium infusion. Mental stress is then performed using the subtraction paradigm described above, and the rubidium generator is started immediately after mental stress is completed. A 7.5-minute stress emission scan in list mode is started immediately after the beginning of rubidium infusion. Lastly, for approximately ½ the patients, pharmacologic stress with Regadenason 0.4 mg will be performed as well, followed by a 3rd 7.5 minute stress. The results of this 3rd scan, which provide coronary flow reserve, will be performed as part of a secondary analysis. Manual rigid-body registration of the CT and PET images is performed by using manufacturer-provided software.

Static Reconstruction. The diagnostic images will be reconstructed using ordered-subsets expectation maximization (OSEM) algorithm (4 iterations, 16 subsets) with a Gaussian post-filter of 7 mm. A standard 2-minute delay will be applied to the list mode data for final attenuation correction static reconstruction.

PET Flow Analysis. *Modeling of Tracer Kinetics for Absolute Blood Flow.* After reconstruction, images are translated into short axis orientation using our in-house software as per normal clinical protocol. Images are transferred to the Emory Cardiac Toolbox (ECTb) software that contains FlowTool for myocardial blood flow calculations. The calculation proceeds without further user interaction. Thirty-two regions of interest (ROIs) are automatically drawn to characterize the uptake in different areas: 9 on the background, 4 concentric rings inside the left ventricle (LV), 2 inside the right ventricle (RV) and one each on the standard 17 segments. The rest and stress images are handled separately. This sampling procedure is performed on a late summed image (2-5 min) when the tissue to blood contrast is high. The ROIs are then transferred to each image to obtain the time activity curves (TACs). MBF is calculated from the TACs using the factor analysis method of El-Fakhri[29] and the kinetic model of Hutchins[30]. Briefly, the factor analysis is used to determine left and right ventricle and myocardial tissue factor curves. (Example results are shown in the above figure.) For each myocardial ROI, the determined contributions from LV and RV blood are subtracted. These spillover corrected curves and the LV factor curve are used as the inputs for kinetic modelling. It is recognized that the activity measured in the tissue ROIs is an underrepresentation of the true activity due to the finite resolution of the scanner and cardiac motion as discussed below.

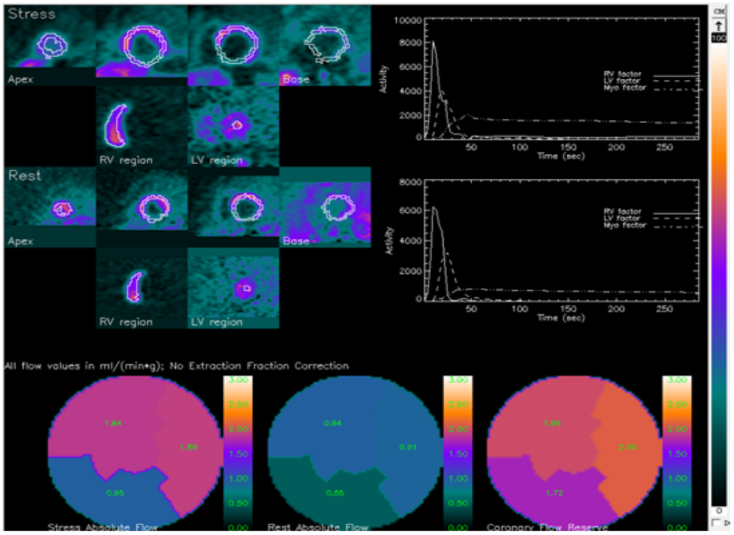

Figure 1. QC display of flow measurements showing stress and rest slices with superimposed sampling region (top left), corresponding RV/LV and myocardial TACS (right) and stress, rest and CFR polar maps with generic vascular territories.

The two-tissue compartment, three parameter model of Hutchins[30] is fit to the data using a time-weighted least-squares figure of merit. The K1 parameter, which represents the rate that tracer moves from the blood to the first tissue compartment, is interpreted as representing flow times extraction (MBF*mEF) where mEF is the myocardial extraction fraction of the tracer. Neither ^{82}Rb nor $^{13}NH_3$ are ideal flow tracers since their mEF is less than 1.0 and depends on flow. At a resting flow of 1 ml/min/g, mEF is approximately 0.5 for ^{82}Rb; at 3.0 ml/min/g after pharmacological stress induction, it falls to approximately 0.3 respectively.[31] mEF correction using Yoshida[31] or Lortie[32] equations is implemented in FlowTool as user selectable options. The user also has the option to apply a partial volume correction by applying a general recovery coefficient[33] to the myocardial tissue.

MBF and derived MFR are output on the current FlowTool display (Fig 1). Note that general implementation of versions of our FlowTool has been used in several clinical investigations. In addition, conventional measurements of LV function (EF, EDV, ESV, mass, thickening, dyssynchrony) will be performed as part of the ECTb processing.[34] Coronary flow reserve will be calculated as the ratio of total flow during stress to flow during rest.

Blood tests: Pre-, immediate-post mental stress, and 90 minute post mental stress blood samples will be centrifuged and the plasma stored for measurement of catecholamine levels. In addition samples will be stored for future measurement of markers of inflammation, oxidation and other biomarkers. The same protocols will be performed for all visits.

Microvascular blood flow measurement using finger plethysmography: We have employed a novel device that measures finger pulse wave amplitude responses using a robust modified form of volume plethysmography. Pulsatile arterial tonometry (PAT, Itamar-Medical, Israel) is designed to reflect only pulsatile arterial volume changes. The main features of this device include a proximal probe component and application of a constant counter pressure of 7 mm of mercury within the whole probe to keep venous transmural pressure deliberately negative. These features prevent venous pooling and stasis within the instrumented part of the finger and inhibit blood volume changes. The counter pressure serves to partially unload arterial wall tension, thus improving the range of the arterial pulse excursions. The probe components are connected via thin flexible tubing to isolated volume reservoirs to buffer pressure changes within the probe. A further volume reservoir not connected to the probe serves as a pressure reference. The pressure changes accompanying peripheral volume changes are fed to a personal computer by which the signal is bandpass filtered (0.3 to 30 Hz), amplified, displayed and stored. We will examine the mean PAT ratio, which reflects the magnitude of change in finger-tip blood flow induced by mental stress by measuring the ratio of pulse wave amplitude during mental stress compared to rest. The PAT ratio is expected to be lower during mental stress, consistent with stress-induced vasoconstriction.[35] The assessment of pulse wave amplitude by peripheral arterial tonometry is relatively free of artifacts when individuals are monitored at rest and decreases in pulse wave amplitude are most likely due to microvascular constriction.[36-39]

Endothelial function assessment: Flow-mediated vasodilation (FMD) of the brachial artery: Brachial artery FMD using ultrasonography for determining endothelial function will be utilized before and 30 minutes after mental stress testing which has in previous studies been associated with peak reduction in endothelial function. We will determine endothelium-dependent FMD of the brachial arteries from two-dimensional ultrasound images according to established and validated methodologies. Images will be obtained with an Acuson 10 mHz linear array transducer and an Acuson Aspen ultrasound system. [40-43] We will perform imaging with the subject resting supine for at least 10 minutes on a hospital bed in a quiet setting. For each subject, optimal brachial artery images will be obtained between 2 and 10 cm above the antecubital crease. After baseline measurements a blood pressure cuff is inflated to 60 mm Hg above systolic pressure over the proximal portion of the right arm for 5 minutes. Endothelium-dependent function will be determined during the first two minutes of release of the cuff as previous studies have shown that maximal dilatation occurs 1 minute after cuff deflation.

Endothelium-independent responsiveness to nitroglycerin will not be measured so as to not affect the vascular measurements that follow. Four triggered events (defined as the end of the T wave on the EKG) for each intervention will be recorded and downloaded to an analysis system that allows automatic edge detection of the M-line that defines the intima-media interface for both the near wall and the far wall of the artery using a customized software (Medical Imaging Applications Inc, Iowa). Analysis will be performed by individuals blinded to clinical and laboratory status of the subjects. A linear portion of the vessel will be chosen. Measurements from the twelve frames will be averaged for every intervention. The end point of measurement will be the percent change in diameter in response to reactive hyperemia.

Reproducibility of FMD measurements: The mean difference in FMD (%) between 2 consecutive assessments performed in 11 subjects an average of 8 days apart was 1.26 (±0.76)%, with r=0.75. The mean difference in the FMD (%) between 2 readings of the same 11 measurements was 0.82 (±0.48)%, with r=0.97.

PAT reactive hyperemia index as a measure of vascular endothelial function**:**

Technique: Reactive hyperemia index of the finger will also be measured simultaneously with FMD measurement as described above. With the patients in the supine position in a temperature controlled room with both hands at the same level, PAT (Itamar Inc.) probes, one on each hand and at the same finger (fingers 2, 3 or 4) will be placed (Please also see section C 2 above). The blood pressure cuff will remain on one hand (the study arm) and the other arm will serve as a control. Following the 10-minure equilibration period, the blood pressure cuff is inflated to 60 mmHg above systolic pressure for 5 minutes with continuous recording. This is followed by deflation of the cuff and continuous recording from both arms for 5 minutes. Reactive hyperemia index (RHI) is calculated as a ratio of hyperemic to baseline flow signal.

Measurement of Arterial Stiffness/Compliance: Technique:Vascular function will be assessed as pulse wave velocity and radial pulse wave analysis measured noninvasively using the SphygmoCor® Pulse Wave Velocity system (PWV Medical, NSW, Australia). In brief, peripheral pressure waveforms are recorded from the radial artery at the wrist, using applanation tonometry with a high-fidelity micromanometer. After 20 sequential waveforms have been acquired, a validated generalized transfer function will be used to generate the corresponding central aortic pressure waveform.

The augmentation index: The SphygmoCor is a computerized, portable and simple-to-use device used to assess pulse waveforms. Aortic pulse waveform, augmentation index, and central aortic pressure will be derived at the radial artery by applanation tonometry. Only high-quality recordings, defined as an in-device quality index >80% (derived from an algorithm including average pulse height, pulse height variation, diastolic variation, and the maximum rate of rise of the peripheral waveform) and acceptable curves on visual inspection by the PI will be included in the analysis. Studies with inadequate tracings will be excluded. All pulse wave analyses will be taken in the sitting position in a quiet room after a brief period (at least 15 minutes) of rest.

Augmentation index (Aix) and augmented pressure (AP) will be derived from this with the technique of pulse wave analysis. The merging point of the incident and the reflected wave (the inflection point) is identified on the generated aortic pressure waveform. AP is the maximum systolic pressure minus pressure at the inflection point. The AIx is defined as the AP divided by pulse pressure and expressed as a percentage. Larger values of AIx indicate increased wave reflection from the periphery or earlier return of the reflected wave as a result of increased pulse wave velocity (attributable to increased arterial stiffness) and vice versa. In addition, because AIx is influenced by heart rate, an index normalized for heart rate of 75 bpm (AIx@75) will also be used in accordance with Wilkinson et al. Time to return of the reflected wave (Tr) is the time from the beginning of the derived aortic systolic pressure waveform to the inflection point and can be used as a substitute for pulse wave velocity (a higher pulse wave velocity will lead to a shorter Tr.

<u>Pulse wave velocity measurement:</u> The pulse wave velocity (PWV) system measures the velocity of the blood pressure waveform between any two superficial artery sites. It uses a single-lead electrocardiogram and then a tonometer to measure the pressure pulse waveform sequentially in the two peripheral artery sites. Transcutaneous Doppler flow velocity recordings will be carried out simultaneously at the base of the neck over the common carotid artery and the femoral artery in the groin with the PWV system attached to a laptop computer (SphygmaCor). The time delay (t) will be measured between the feet of the flow waves recorded at these points. The distance (d) traveled by the pulse wave is measured over the body surface as the distance between the two recording sites minus that from the suprasternal notch to the carotid artery recording site. PWV is calculated as PWV=d/t. The reproducibility of the measurement for aortic PWV has been published previously.[44-46]

<u>Reproducibility and experience:</u> We are currently conducting an NIH funded study in a multi-ethnic community population of 1000 subjects who are all having arterial compliance assessed in this manner. Three technicians have been trained to conduct these measurements reliably and reproducibly. The mean difference between consecutive AIx and AP measurements performed on 2 different days of 1.37% and 1.2 mm Hg, respectively; 95% limits of agreement for AIx and AP were 10.1% and 9.6 mm Hg, respectively.

<u>Autonomic Measurements</u>: We will assess measures of sympathetic and parasympathetic tone to acute mental stress using the VU AMS holter device (VU University, Netherlands) and plasma catecholamines. The VU AMS device measures ECG and impedance cardiography simultaneously, which allows for the measurement of the pre-ejection period (PEP), a measure of cardiac sympathetic function. PEP is quantified as the difference in time between R wave peak and the opening of the aortic valves. More details on the exact methods used to calculate PEP using the VU AMS device are described elsewhere.[47] For parasympathetic tone, high frequency heart rate variability will be calculated by performing the Fast Fourier transform, and respiratory sinus arrhythmia will also be calculated as previously described using the VU DAMS software.[47] Additionally, plasma catecholamine measurements will include dopamine, norepinephrine, and epinephrine measurements. Plasma catecholamines will be assayed prior to and post stress. The 3-CAT high sensitivity kit will be used according to the manufacturer's protocol (Rocky

Mountain Diagnostics, Colorado Springs, CO). This method has high sensitivity (limit of detection 25 pg/ml for NE), analytical specificity, and reproducibility (CVs <10%).

Participant selection

We will recruit up to 30 subjects in order for 24 subjects to complete the study from the pool of >600 CAD patients from MIPS, all from Emory University-affiliated hospitals or clinics who have undergone rest/stress SPECT with mental stress. The inclusion and exclusion criteria have been published elsewhere.[48] These patient have previously consented for future studies. Patients with previously demonstrated mental stress ischemia will be preferentially recruited, as such patients will likely benefit the most from the biofeedback intervention.

Statistical analysis

In both intervention and control groups, we will calculate changes in MBF and myocardial flow reserve (MFR) with mental stress between baseline and the end of the protocol, and test whether the differences are different from zero. We will then compare the changes in MBF and MFR between the two randomization groups using mixed model regression analysis. If values are not normally distributed, appropriate transformations will be employed. If the two groups are not balanced for major characteristics despite randomization (demographic factors, comorbidities, lifestyle factors, etc.), these factors will be adjusted for in the analysis.

Power Calculations

The table shows a range of sample sizes needed to achieve a power of 0.8-0.9 with an effect size of 25% to 50% improvement in MFR (an improvement of 25% would be deemed clinically significant). A sample size of 20 would achieve at least 80% power with an effect size of 25%. Because some patients may drop out, we plan to increase the sample size by 20%, bringing the final sample size to 24, with 16 in the Biofeedback group and 8 in the Control group.

Table. Sample size determination and power.
(BF=Biofeedback, C=Control)

BF Group MFR	Control Group MFR	SD	Power	Improvement in MFR	Total n
1.38	1.1	0.2	0.8	25%	20
1.65	1.1	0.2	0.8	50%	8
1.38	1.1	0.2	0.9	25%	26
1.65	1.1	0.2	0.9	50%	10

Adverse event reporting

All adverse events (AEs) will be graded as to their attribution (unrelated to protocol, or possibly, probably, or definitely related to protocol). Any AE that is reported by a study subject or by medical staff caring for the subject and which meets the criteria will be documented as such.

Serious adverse events (SAEs) are defined as: any experience that suggests a significant hazard, such as events which: a) are fatal, b) are life threatening, c) result in persistent or significant disability, d) requires or prolongs inpatient hospitalization, e) results in congenital

anomalies/birth defects, or f) in the opinion of the investigators represents other significant hazards or potentially serious harm to research subjects or others. In the case of a severe adverse event a code will be called to access immediate emergency medical attention. This adverse event will be immediately communicated to the research coordinator. Any SAE will be reported to the PI and the IRB within 24 hrs.

Unanticipated problems (UPs) are defined as unanticipated events involving risks to participants or others that are possibly, probably, or definitely related to the research; or anticipated events that occur with a greater frequency, duration, or severity than what is documented in the protocol, informed consent, or investigator's brochure; or other unanticipated information that changes the risk benefit ratio or that indicates participants or others might be at greater risk of harm than was previously known. SAEs and UPs are to be reported to the supervising institutional IRB and sponsor as per their policies and procedures.

The investigators and staff will track and summarize AE frequency, severity, and relatedness at a frequency appropriate to insure subject safety.

Potential ("expected") adverse events and plan for detecting problems and minimizing subject risk during this trial: (1) PET radiation exposure, (2) intravenous catheter, (3) blood draw, (4) mental stress test, (5) inconvenience of wearing EKG leads.

Expected adverse events will be detailed in the Consent Form and include the following: (1) PET radiation exposure, (2) intravenous catheter, (3) blood draw, (4) mental stress test, (5) inconvenience of wearing EKG leads.

Procedures for minimizing risks: (1) A radiation exposure history will be obtained from each subject prior to their being exposed to the PET scan. Every effort will be made to keep the exposure to a minimum. (2) Medication can be given prior to the PET scan to help with apprehension and anxiety if necessary. A physician will be present throughout the procedure and vital signs and heart rate will be monitored while the Rb^{82} dose is infusing. (3) Every effort will be made to insert the IV catheter and perform blood draws as painlessly as possible on the first attempt. (4) Immediately after the mental stress test, study staff will debrief subjects; if necessary the study psychiatrist will see the patient and take further action. Patients who become suicidal will be escorted to the emergency room. Arrangements for treatment referral will be made during the course of the study if necessary. (5) The adhesive back of the monitor leads could cause some irritation to the skin. The irritation can be relieved by hydrocortisone cream and will usually disappear in 1-2 days even with no treatment.

Data Safety Monitoring Plan (DSMP)

The Data Safety Monitoring Plan (DSMP) outlined below will adhere to the protocol approved by the institutional IRB responsible for study oversight. An IRB-approved written informed consent will be obtained from each subject at entry into the study; elements of informed consent will include: (a) having the subject review the study consent form; (b) having the investigator(s) or study staff meet with the subject to review the consent, confirm understanding, and answer any questions; and (c) once the investigator(s) or study staff are convinced that the protocol is understood and that there is agreement to participate, having the consent signed. A copy of the signed consent form will be provided to subject and/or their legally authorized representative. Documentation of the informed consent process will be made in the research and/or medical records as appropriate. The principal investigator (PI) will review all data collection forms for

completeness and accuracy of the data as well as protocol compliance. Any data inconsistencies will be resolved by study staff as soon as possible. The PI will review this protocol on a continuing basis for subject safety and protocol deviations or study noncompliance. Reportable Events will be communicated to the local institutional IRB as required. The results of the review will also be included in progress reports submitted to the local institutional IRB, or any other regulatory agency or sponsor, as required.

Stopping Rules for Mental Stress Testing.[49]

Absolute Indications for Termination

- ST-segment elevation (>1.0 mm) in leads without Q waves (other than V_1 or aVR).
- Drop in systolic blood pressure >10 mm Hg (persistently below baseline), despite an increase in workload, when accompanied by any other evidence of ischemia.
- Moderate-to-severe angina at a level that the patient wishes to stop.
- Central nervous system symptoms (eg, ataxia, dizziness, or near syncope).
- Signs of poor perfusion (cyanosis or pallor).
- Sustained ventricular tachycardia.
- Technical difficulties monitoring the ECG or systolic blood pressure.
- Subject's request to stop.

Relative Indications for Termination

- ST or QRS changes such as excessive ST displacement (horizontal or downsloping of >2 mm) or marked axis shift.
- Drop in systolic blood pressure >10 mm Hg (persistently below baseline) despite an increase in workload, in the absence of other evidence of ischemia.
- Increasing chest pain.
- Fatigue, shortness of breath, wheezing, leg cramps, or claudication.
- Arrhythmias other than sustained ventricular tachycardia, including multifocal ectopic, ventricular triplets, supraventricular tachycardia, heart block, or bradyarrhythmias.
- General appearance (see below).
- Hypertensive response (systolic blood pressure >250 mm Hg)
- Development of bundle-branch block that cannot be distinguished from ventricular tachycardia.
- If the patient becomes excessively anxious or uncomfortable, or requests us to stop.

Stopping Rules for Study

Absolute indications for study wide termination (determined by the PI)

- the intervention is associated with adverse effects that call into question the safety of the intervention
- difficulty in study recruitment or retention that will significantly impact the ability to evaluate the study endpoints
- any new information becomes available during the trial that necessitates stopping the trial
- other situations occur that might warrant stopping the trial

Plans for transmission of temporary or permanent suspension actions: Any actions that mandate temporary or permanent suspension of study will be transmitted to the institutional IRB, and, if appropriate, to the study sponsor, FDA, or the National Institutes of Health.

Plans for protecting subject confidentiality: All information and materials that are obtained for research purposes only will be kept in strict confidence. Confidentiality will be assured by the use of subject codes rather than personal identifiers. No names will be used on study forms. A crosswalk linking will be kept under lock and key in another location. After the study is completed, the crosswalk will be destroyed. The study database will be secured, and information will only be entered using subject identifier codes rather than personal identifiers. Electronic communication will involve only coded, unidentifiable information.

Plans for assuring data accuracy and protocol human safety compliance: An IRB-approved written informed consent will be obtained from each subject at entry into the study; elements of informed consent will include: (a) having the subject review the study consent form; (b) having the investigator(s) or study staff meet with the subject to review the consent, confirm understanding, and answer any questions; and (c) once the investigator(s) or study staff are convinced that the protocol is understood and that there is agreement to participate, having the consent signed. A (bi)weekly meeting with the staff, Study Coordinator and the PI is held to discuss any issues that may arise. A review of data collection forms is done for accuracy and protocol compliance. Data collection forms are scanned and verified for accuracy before storing in database. The PI will review this protocol on a continuing basis for subject safety and include the results of the review in annual progress reports submitted to the IRB. Patient Monitoring: will be performed by the PI, the Co-Investigators, and the Research Coordinator(s). Any unanticipated events are reported to the IRB in a timely manner per protocol.

The above detailed plans should assure data accuracy and protocol human safety compliance for this study. These include computerized database management, and IRB oversight and communication. This plan, together with oversight by the IRB, should be sufficient for studies that do not require a DSMB.

References

1. Lehrer PM, Vaschillo E, Vaschillo B, et al. Heart rate variability biofeedback increases baroreflex gain and peak expiratory flow. *Psychosom Med.* 2003;65(5):796-805.
2. McCraty R, Barrios-Choplin B, Rozman D, Atkinson M, Watkins AD. The impact of a new emotional self-management program on stress, emotions, heart rate variability, DHEA and cortisol. *Integr Physiol Behav Sci.* 1998;33(2):151-170.
3. Lin G, Xiang Q, Fu X, et al. Heart Rate Variability Biofeedback Decreases Blood Pressure in Prehypertensive Subjects by Improving Autonomic Function and Baroreflex. *Journal of Alternative & Complementary Medicine.* 2012;18(2):143.
4. Steptoe A, Kivimaki M. Stress and cardiovascular disease. *Nature reviews. Cardiology.* 2012;9(6):360-370.
5. Strike PC, Steptoe A. Systematic review of mental stress-induced myocardial ischaemia. *Eur Heart J.* 2003;24(8):690-703.
6. Holmes SD, Krantz DS, Rogers H, Gottdiener J, Contrada RJ. Mental stress and coronary artery disease: a multidisciplinary guide. *Prog Cardiovasc Dis.* 2006;49(2):106-122.
7. Jain D, Burg M, Soufer R, Zaret BL. Prognostic implications of mental stress-induced silent left ventricular dysfunction in patients with stable angina pectoris. *Am J Cardiol.* 1995;76(1):31-35.
8. Jiang W, Babyak M, Krantz DS, et al. Mental stress--induced myocardial ischemia and cardiac events. *JAMA.* 1996;275(21):1651-1656.
9. Sheps DS, McMahon RP, Becker L, et al. Mental stress-induced ischemia and all-cause mortality in patients with coronary artery disease: Results from the Psychophysiological Investigations of Myocardial Ischemia study. *Circulation.* 2002;105(15):1780-1784.
10. Yeung AC, Vekshtein VI, Krantz DS, et al. The effect of atherosclerosis on the vasomotor response of coronary arteries to mental stress. *N Engl J Med.* 1991;325(22):1551-1556.
11. Dakak N, Quyyumi AA, Eisenhofer G, Goldstein DS, Cannon RO, 3rd. Sympathetically mediated effects of mental stress on the cardiac microcirculation of patients with coronary artery disease. *Am J Cardiol.* 1995;76(3):125-130.
12. Hedetoft M, Olsen NV. Evaluation of endothelial function by peripheral arterial tonometry and relation with the nitric oxide pathway. *Nitric Oxide.* 2014;42:1-8.
13. Burg MM, Graeber B, Vashist A, et al. Noninvasive detection of risk for emotion-provoked myocardial ischemia. *Psychosom Med.* 2009;71(1):14-20.
14. Hassan M, Li Q, Brumback B, et al. Comparison of peripheral arterial response to mental stress in men versus women with coronary artery disease. *The American journal of cardiology.* 2008;102(8):970-974.
15. Hassan M, York KM, Li H, et al. Usefulness of peripheral arterial tonometry in the detection of mental stress-induced myocardial ischemia. *Clinical cardiology.* 2009;32(9):E1-6.
16. Arrighi JA, Burg M, Cohen IS, et al. Myocardial blood-flow response during mental stress in patients with coronary artery disease. *Lancet.* 2000;356(9226):310-311.
17. Dimsdale JE. Psychological stress and cardiovascular disease. *J Am Coll Cardiol.* 2008;51(13):1237-1246.
18. Blumenthal JA, Jiang W, Babyak MA, et al. Stress management and exercise training in cardiac patients with myocardial ischemia. Effects on prognosis and evaluation of mechanisms. *Arch Intern Med.* 1997;157(19):2213-2223.
19. Blumenthal JA, Babyak M, Wei J, et al. Usefulness of psychosocial treatment of mental stress-induced myocardial ischemia in men. *Am J Cardiol.* 2002;89(2):164-168.

20. Blumenthal JA, Sherwood A, Babyak MA, et al. Effects of exercise and stress management training on markers of cardiovascular risk in patients with ischemic heart disease: a randomized controlled trial. *JAMA.* 2005;293(13):1626-1634.
21. Linden W, Phillips MJ, Leclerc J. Psychological treatment of cardiac patients: a meta-analysis. *Eur Heart J.* 2007;28(24):2972-2984.
22. Bradley RT, Galvin P, Atkinson M, Tomasino D. Efficacy of an Emotion Self-regulation Program for Promoting Development in Preschool Children. *Glob Adv Health Med.* 2012;1(1):36-50.
23. Lemaire JB, Wallace JE, Lewin AM, de Grood J, Schaefer JP. The effect of a biofeedback-based stress management tool on physician stress: a randomized controlled clinical trial. *Open Med.* 2011;5(4):e154-163.
24. McCraty R, Deyhle A, Childre D. The global coherence initiative: creating a coherent planetary standing wave. *Glob Adv Health Med.* 2012;1(1):64-77.
25. Boger LA, Volker LL, Hertenstein GK, Bateman TM. Best patient preparation before and during radionuclide myocardial perfusion imaging studies. *Journal of Nuclear Cardiology.* 2006;13(1):98-110.
26. Shaw LJ, Mieres JH. The role of noninvasive testing in the diagnosis and prognosis of women with suspected CAD. *J Fam Pract.* 2005;Suppl:4-5, 7.
27. Mahmarian JJ, Dakik HA, Filipchuk NG, et al. An initial strategy of intensive medical therapy is comparable to that of coronary revascularization for suppression of scintigraphic ischemia in high-risk but stable survivors of acute myocardial infarction. *J Am Coll Cardiol.* 2006;48(12):2458-2467.
28. Soufer R, Bremner JD, Arrighi JA, et al. Cerebral cortical hyperactivation in response to mental stress in patients with coronary artery disease. *Proc Natl Acad Sci U S A.* 1998;95(11):6454-6459.
29. El Fakhri G, Sitek A, Guerin B, Kijewski MF, Di Carli MF, Moore SC. Quantitative dynamic cardiac 82Rb PET using generalized factor and compartment analyses. *J Nucl Med.* 2005;46(8):1264-1271.
30. Hutchins GD, Caraher JM, Raylman RR. A region of interest strategy for minimizing resolution distortions in quantitative myocardial PET studies. *J Nucl Med.* 1992;33(6):1243-1250.
31. Yoshida K, Mullani N, Gould KL. Coronary flow and flow reserve by PET simplified for clinical applications using rubidium-82 or nitrogen-13-ammonia. *J Nucl Med.* 1996;37(10):1701-1712.
32. Lortie M, Beanlands RS, Yoshinaga K, Klein R, Dasilva JN, DeKemp RA. Quantification of myocardial blood flow with 82Rb dynamic PET imaging. *Eur J Nucl Med Mol Imaging.* 2007;34(11):1765-1774.
33. Kessler RM, Ellis JR, Jr., Eden M. Analysis of emission tomographic scan data: limitations imposed by resolution and background. *J Comput Assist Tomogr.* 1984;8(3):514-522.
34. Garcia EV, Faber TL, Cooke CD, Folks RD, Chen J, Santana C. The increasing role of quantification in clinical nuclear cardiology: the Emory approach. *J Nucl Cardiol.* 2007;14(4):420-432.
35. Hassan M, Li H, Li Q, Lucey DG, Fillingim RB, Sheps DS. Usefulness of Peripheral Arterial Tonometry in the Detection of Mental Stress-induced Myocardial Ischemia. *Clinical Cardiology.* 2009;in press.
36. Burton A. The range and variability of the blood flow in the human fingers and the vasomotor regulation of body temperature. *Amer J Physiology.* 1939;127:437-453.
37. Wilkins R, Doupe, J, Newman, HW. . The rate of blood flow in normal fingers. *Clinical Science.* 1938;3:403-411.
38. Schnall RP, Shlitner A, Sheffy J, Kedar R, Lavie P. Periodic, profound peripheral vasoconstriction--a new marker of obstructive sleep apnea. *Sleep.* 1999;22(7):939-946.
39. Lavie P, Schnall RP, Sheffy J, Shlitner A. Peripheral vasoconstriction during REM sleep detected by a new plethysmographic method. *Nat Med.* 2000;6(6):606.

40. Bonetti PO, Barsness GW, Keelan PC, et al. Enhanced external counterpulsation improves endothelial function in patients with symptomatic coronary artery disease. *J Am Coll Cardiol.* 2003;41(10):1761-1768.
41. Verma S, Buchanan MR, Anderson TJ. Endothelial Function Testing as a Biomarker of Vascular Disease. *Circulation.* 2003;108(17):2054-2059.
42. Gerhard MD, Knab S, Delagrange D, et al. Close Relation of Endothelial Function in the Human Coronary and Peripheral Circulations. *Journal of the American College of Cardiology.* 1995;26(5 SU -):1235-1241.
43. Cohn JN, Quyyumi AA, Hollenberg NK, Jamerson KA. Surrogate markers for cardiovascular disease: functional markers. *Circulation.* 2004;109(25 Suppl 1):IV31-46.
44. Pauca AL, O'Rourke MF, Kon ND. Prospective evaluation of a method for estimating ascending aortic pressure from the radial artery pressure waveform. *Hypertension.* 2001;38(4):932-937.
45. Siebenhofer A, Kemp C, Sutton A, Williams B. The reproducibility of central aortic blood pressure measurements in healthy subjects using applanation tonometry and sphygmocardiography.[comment]. *Journal of Human Hypertension.* 1999;13(9):625-629.
46. Savage MT, Ferro CJ, Pinder SJ, Tomson CR. Reproducibility of derived central arterial waveforms in patients with chronic renal failure. *Clinical Science.* 2002;103(1):59-65.
47. Houtveen JH, Groot PF, Geus EJ. Effects of variation in posture and respiration on RSA and pre-ejection period. *Psychophysiology.* 2005;42(6):713-719.
48. Ramadan R, Sheps D, Esteves F, et al. Myocardial Ischemia During Mental Stress: Role of Coronary Artery Disease Burden and Vasomotion. *Journal of the American Heart Association: Cardiovascular and Cerebrovascular Disease.* 2013;2(5):e000321.
49. Fletcher GF, Balady GJ, Amsterdam EA, et al. Exercise standards for testing and training: A statement for healthcare professionals from the American Heart Association. *Circulation.* 2001;104(14):1694-1740.
